# Baseline Hepatitis B Surface Antibody (Anti-HBs) Titers in Healthcare Workers at a Tertiary Care Center in Chennai, India

**DOI:** 10.7759/cureus.88973

**Published:** 2025-07-29

**Authors:** Vaishnavi Konda, Shanti Vijayaraghavan

**Affiliations:** 1 Department of Hepatology, Sri Ramachandra Medical College, Chennai, IND

**Keywords:** anti-hbs, anti- hbs titers, healthcare workers, hepatitis b vaccine, viral hepatitis b

## Abstract

Background

Healthcare workers (HCWs) are at increased risk of hepatitis B virus (HBV) infection due to occupational exposure to blood and body fluids. Despite the availability of effective vaccination, adherence to vaccination recommendations among HCWs remains suboptimal in many healthcare settings.

Objective

To determine baseline hepatitis B surface antibody (anti‑HBs) titers among HCWs at a tertiary hospital.

Methods

A cross-sectional study was conducted involving 1,909 HCWs from a tertiary hospital. Data collected included age, gender, and work area. Anti‑HBs titers were quantitatively measured using the ARCHITECT Anti‑HBs test (Abbott Laboratories, Illinois, USA). Statistical analyses, including chi-square tests, were conducted to evaluate the association between demographic variables and vaccine response.

Results

The majority of participants (43%) belonged to the 16-25-year age group, and 62.85% were from the nursing department. The overall vaccine response rate was 93.0%. Vaccine response was highest among individuals aged 26-35 years (95.6%) and lowest in those aged 56-75 years (82.1%). Statistically significant associations were found between response rates and age, sex, and work area (p < 0.001 for all) using chi-square analysis.

Conclusion

Our findings indicate that most HCWs developed a protective response to the hepatitis B vaccine (93.0%), particularly those aged 26 to 35 years (95.6%). Nonetheless, the existence of a 7.0% non-responder group emphasizes the need for additional follow-up. Age, sex, and occupational role were significantly associated with antibody response, suggesting that individualized immunization strategies may be beneficial. Ensuring immunity in non-responders through appropriate re-vaccination could play a key role in closing immunity gaps among HCWs.

## Introduction

Chronic hepatitis B virus (HBV) infection carries significant morbidity and mortality, as 15%-25% of cases may progress to cirrhosis or liver cancer [1,2]. Medical personnel and students are at increased risk, largely due to occupational exposures such as needlestick injuries. In Southeast Asia, up to 46.6% of nurses in developing countries have reported experiencing such injuries. In India, the precise incidence of needlestick injuries is not well documented due to a lack of comprehensive data [3]. Current guidelines recommend that healthcare providers undergo testing for hepatitis B surface antibody (anti‑HBs) one to two months after completing their vaccination schedule to confirm immunity [4]. Vaccine effectiveness is indicated by the presence of anti‑HBs, reflecting the activation of memory T cells. Anti‑HBs titer of 10 mIU/mL, measured one to two months post‑vaccination, is widely accepted as a reliable marker of protection against HBV infection [5]. For healthcare workers (HCWs) with anti‑HBs titers below 10 mIU/mL, a subsequent vaccine dose is recommended, followed by antibody level testing after one to two months. Following this additional dose, 15% to 25% of individuals typically exhibit an adequate antibody response. If the anti‑HBs titer remains below 10 mIU/mL after this repeat dose, two further doses should be administered, with serologic testing conducted two months thereafter. Approximately 50% of individuals will achieve a sufficient antibody response after these three extra doses. If the anti‑HBs level remains below 10 mIU/mL, the HCW is classified as a non‑responder to the vaccine and should receive post‑exposure prophylaxis.

Furthermore, non-responders should be tested for HBsAg and total anti-HBc to determine their infectious status [6]. Lower seroconversion rates have been associated with factors such as increasing age, higher levels of immunocompromise, smoking, and elevated body mass index [7-10]. Identifying vaccine non‑responders is critical due to their increased susceptibility to HBV infection. However, there is a paucity of data in India regarding anti‑HBs titers in HCWs. This study aimed to evaluate baseline anti‑HBs titers in HCWs and to assess the influence of age, sex, and occupation on these levels. The strength of our study lies in its inclusion of paramedical and administrative staff, often underrepresented in research despite their potential occupational risk of HBV transmission. This research addresses the data gap specific to India, providing valuable insights into baseline anti‑HBs titers and their variability among HCWs, including nursing, paramedical, and administrative personnel, in a tertiary care hospital.

## Materials and methods

Study design, setting, and participants

This was a cross-sectional observational study conducted at Sri Ramachandra Medical College and Hospital, a tertiary-care hospital in Chennai in 2024. A total of 1,909 HCWs, who included nurses, paramedical staff, and administrative staff, participated in the study. Adults aged over 18 years working at the hospital who provided written informed consent were eligible. We excluded HCWs who tested positive for hepatitis B surface antigen (HBsAg). We collected non-identifiable demographic data, including age, gender, and work area-while ensuring that all other personal details remained fully anonymized. Anti-HBs titers were measured using the ARCHITECT Anti-HBs test (Abbott Laboratories, Illinois, USA). Ethics Committee of Sri Ramachandra Medical College and Hospital issued approval CSP-MED/23/JUL/89/156.

Sample collection and laboratory processing 

Under aseptic conditions, 3 mL of venous blood was drawn via venipuncture from each participant. The blood samples were then centrifuged at 2000 rpm for 20 minutes to separate the serum, which was stored at −20°C until the anti-HBs test was conducted. The ARCHITECT Anti-HBs test, which utilizes chemiluminescent microparticle immunoassay (CMIA) technology, was utilized to quantitatively measure antibodies against the HBsAg in the serum samples. The ARCHITECT Anti-HBs assay is a two-step procedure that relies on CMIA technology. In the first step, the specimen diluent containing recalcified human plasma is mixed with recombinant HBsAg (rHBsAg)-coated paramagnetic microparticles. Any anti-HBs in the sample attach to the rHBsAg-coated microparticles. Following a washing step with a phosphate-buffered saline (PBS) solution, a rHBsAg conjugate labeled with acridinium is introduced. In the second step, the reaction mixture undergoes another washing cycle, after which a pre-trigger solution containing 1.32% (w/v) hydrogen peroxide and a trigger solution with 0.35 N sodium hydroxide are added. The chemiluminescent reaction produced is quantified in relative light units (RLUs). The level of chemiluminescence is directly related to the concentration of anti-HBs in the sample. The anti-HBs concentration is then calculated using a previously established calibration curve created by the ARCHITECT system.

The study population was categorized based on the anti-HBs titers as follows: non-responders: anti-HBs titer < 10 mIU/mL; responders: anti-HBs titer ≥ 10 mIU/mL.

Data analysis

The statistical analysis methods used in the study included cross-tabulation and the chi-square test. Cross-tabulation was employed to assess the relationship between categorical variables, and the chi-square test was used to evaluate the statistical significance of these associations. A p-value of < 0.05 was considered statistically significant.

## Results

**Figure 1:**
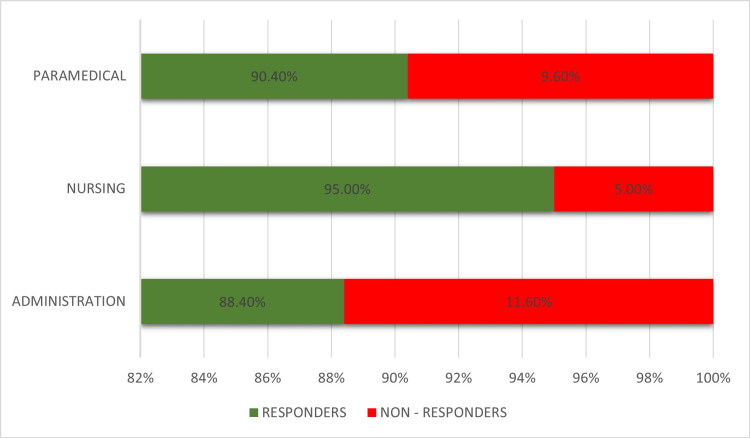
Response rates among different hospital staff categories Bar chart showing the percentage of responders and non-responders among paramedical, nursing, and administrative staff. Responders (green): Paramedical: 90.4%, Nursing: 95.0%, Administration: 88.4%; Non-responders (red): Paramedical: 9.6%, Nursing: 5.0%, Administration: 11.6% All percentages represent the proportion of each group that responded or did not respond to the study survey.

The study population comprised 1,909 HCWs, with the largest age group being 16-25 years (43%) and the smallest 56-75 years (2%). A significant majority (62.85%) of participants were from the nursing department, while the administration department represented the smallest proportion (12.6%). Table 1 presents the distribution of hepatitis B antibody responders and non‑responders across three work areas among 1,909 employees. Nurses exhibited the highest response rate (95.0%), followed by paramedical staff (90.4%) and administrative staff (88.4%), resulting in an overall antibody response rate of 93.0% (Figure 1). The chi-square results show a statistically significant association between work area and vaccine response (p = 0.001), indicating that work area significantly influences vaccine responder status, as shown in Table 2.

**Table 1:** Hepatitis B antibody response by work area (N = 1909)

Work area	Total (n)	Responders (n, %)	Non-responders (n, %)
Administrative staff	241	213 (88.4%)	28 (11.6%)
Nurses	1199	1139 (95%)	60 (5.0%)
Paramedical staff	469	429 (90.4%)	45 (9.6%)
Total	1909	1776 (93.0%)	133 (7.0%)

**Table 2:** Chi‑square analysis of vaccine response by work area Results reflect that no expected cell frequency was below 5 (0.0% of cells), with a minimum expected count of 16.79, confirming that the chi‑square approximation is valid.

Test	Value	df	P-value
Pearson χ²	20.168	2	0.001
Likelihood ratio	19.147	2	0.001
Number of valid cases (N)	1909	–	–

Overall, 93.0% of the study population demonstrated a vaccine response, with 7.0% identified as non-responders. Vaccine response rates varied significantly across age groups (p < 0.001), with the highest rate observed in the 26-35 years group (95.6%) and the lowest in the 56-75 years group (82.1%). A statistically significant difference in response rates was also noted between sexes (p < 0.001), with a higher proportion of males (11.3%) classified as non-responders compared to females (5.7%), as shown in Table 3.

**Table 3:** Chi‑square analysis of vaccine response by sex Results reflect that no expected cell count fell below 5 (0.0% of cells), with the smallest expected count being 30.10, satisfying the assumption for using the chi‑square test of independence.

Test	χ²	df	p
Pearson χ²	19.61	2	0.001
Likelihood ratio	17.94	2	0.001
Total valid cases (N)	1909	—	—

In conclusion, this study of 1,909 HCWs demonstrated an overall vaccine response rate of 93.0% and a non-responder rate of 7.0%. Statistically significant associations were identified between vaccine response and age, sex, and work area (p < 0.001 for all factors).

## Discussion

HBV infection is a significant occupational hazard for HCWs due to routine exposure to blood and potentially infectious body fluids [11]. The primary routes of transmission are percutaneous injuries, particularly needlestick injuries involving contaminated sharps, and mucocutaneous exposures where infected materials contact mucous membranes or broken skin [11]. While needlestick injuries are commonly recognized, many HBV infections among HCWs cannot be linked to specific incidents, suggesting transmission via inapparent exposures, such as contact with contaminated surfaces followed by skin or mucosal inoculation [12]. Outbreaks in settings like hemodialysis units before the widespread use of HBV vaccination highlighted the ease of transmission and emphasized the need for preventive strategies [13]. Epidemiological studies consistently show a strong correlation between blood contact frequency and HBV prevalence among HCWs. Those involved in invasive procedures or blood handling (e.g., surgeons, lab technicians, blood bank personnel) are at greater risk.

HBV is environmentally stable, surviving for at least seven days on surfaces at room temperature, contributing to indirect transmission [11]. HBV infection can manifest as acute hepatitis or progress to chronic infection, increasing the risk of cirrhosis and hepatocellular carcinoma (HCC) [11]. Before vaccination programs, HBV caused significant global morbidity and mortality, contributing to hundreds of thousands of deaths annually [13]. Prior to vaccination, prevention relied on standard infection control and post-exposure prophylaxis with hepatitis B immune globulin (HBIG), which offers only temporary passive immunity and must be administered promptly to be effective [14].

The infectivity of a source patient is heavily influenced by their hepatitis B e-antigen (HBeAg) status. HBeAg positivity correlates with high HBV DNA levels and increased transmissibility [14]. Needlestick injuries from HBeAg-positive sources carry a substantially higher risk of infection, up to 100 times more than HIV, especially in non-immune individuals [11]. HBeAg positivity thus signals high infectivity and active viral replication [15].

The development and implementation of HBV vaccines, first plasma-derived and then recombinant, marked a public health milestone. Vaccination significantly reduced HBV incidence in both HCWs and the general population. Although the vaccine is highly effective, protective anti-HBs titers may decline over time in some individuals [13]. The immune response varies due to age, health status, and genetic factors. Vaccine composition, dosage, and adjuvants (e.g., CpG 1018 in Heplisav-B) also influence immunogenicity [16,17]. Genetic polymorphisms, especially in the human leukocyte antigen (HLA) system and cytokine-encoding genes, affect vaccine response [18,19]. Individuals with conditions like diabetes, hepatitis C, autoimmune disorders, and chronic kidney disease (particularly those on hemodialysis) often exhibit poor vaccine response due to immune system impairment. Antibody titers peak shortly after vaccination and may decline, though immunological memory often provides lasting protection [20]. Monitoring anti-HBs titers helps identify non-responders or those with waning immunity, particularly in high-risk settings. A titer ≥ 10 mIU/mL is considered protective. The Advisory Committee on Immunization Practices (ACIP) recommends serologic testing one to two months post-vaccination for HCWs at risk [21]. Immunocompetent individuals with adequate initial titers generally do not require boosters, even if titers decline. However, immunocompromised individuals, such as those on dialysis, may need periodic boosters to maintain protective levels. For HCWs without documented immunity, serologic testing at the time of employment is advisable to determine immune status and need for revaccination [21].

In our study of 1,909 HCWs, 93% (1776 individuals) were responders and 7% (133) were non-responders, aligning with the findings of Basireddy et al. [22]. A study by Shin et al. in Mumbai found 82.2% responders and 13.8% non-responders [23]. Another Mumbai-based study showed 80.6% achieved titers > 10 mIU/mL post-vaccination [24]. A study in India reported a 96% seroconversion rate [25], while research in Kerala found 92.5% of vaccinated HCWs had adequate titers [26]. Our findings show decreasing anti-HBs titers with advancing age, consistent with other studies, suggesting older individuals may benefit from booster doses. Males had a higher non-responder rate (11.3%) compared to females (5.7%), supporting evidence of stronger immune responses in females, possibly due to hormonal and genetic influences [27,28]. Among job categories, nursing staff showed the highest response rate (95%), likely due to better vaccine compliance and higher awareness of HBV risks. Paramedical staff had a 90.4% response rate, while the administrative group had the lowest at 88.4%. This variation may reflect differences in direct patient contact, perceived exposure risk, and role-specific emphasis on vaccination. Our findings demonstrated a statistically significant relationship between work area and anti-HBs titers, aligning with earlier research [29]. This highlights the role of occupational exposure in shaping vaccine responsiveness and supports the implementation of role-specific vaccination and surveillance protocols. In conclusion, these results emphasize the need for individualized hepatitis B vaccination approaches and routine post-vaccination antibody assessment among HCWs. Prioritizing high-risk groups is essential to maintain long-term immunity and to mitigate the professional health risks associated with HBV.

Our study has certain limitations; being a single-center study with a relatively small sample size, the findings may not be generalizable to a wider population. Additionally, the exclusion of doctors from the study population may have introduced selection bias, potentially affecting the representativeness of the results.

## Conclusions

In summary, the study identified significant correlations between baseline anti-HBs titers and variables such as age, gender, and occupational category. Participants aged 26-35 years demonstrated the highest rates of seropositivity, whereas older individuals exhibited comparatively lower antibody levels. Notably, nursing staff showed the highest positivity rates, possibly reflecting greater awareness of vaccination and increased exposure risk. These results underscore the relevance of routine anti-HBs titer monitoring and suggest that revaccination may be warranted in individuals with declining immunity.
